# Genome Wide Association Identifies *PPFIA1* as a Candidate Gene for Acute Lung Injury Risk Following Major Trauma

**DOI:** 10.1371/journal.pone.0028268

**Published:** 2012-01-25

**Authors:** Jason D. Christie, Mark M. Wurfel, Rui Feng, Grant E. O'Keefe, Jonathan Bradfield, Lorraine B. Ware, David C. Christiani, Carolyn S. Calfee, Mitchell J. Cohen, Michael Matthay, Nuala J. Meyer, Cecilia Kim, Mingyao Li, Joshua Akey, Kathleen C. Barnes, Jonathan Sevransky, Paul N. Lanken, Addison K. May, Richard Aplenc, James P. Maloney, Hakon Hakonarson, for the Trauma ALI SNP Consortium (TASC) investigators

**Affiliations:** 1 Division of Pulmonary, Allergy, and Critical Care Medicine, Department of Medicine, University of Pennsylvania School of Medicine, Philadelphia, Pennsylvania, United States of America; 2 Department of Biostatistics and Epidemiology, Center for Clinical Epidemiology and Biostatistics, University of Pennsylvania School of Medicine, Philadelphia, Pennsylvania, United States of America; 3 Division of Pulmonary and Critical Care Medicine, Department of Medicine, Harborview Medical Center, University of Washington, Seattle, Washington, United States of America; 4 Department of Surgery, Harborview Medical Center, University of Washington, Seattle, Washington, United States of America; 5 Division of Human Genetics, Center for Applied Genomics, The Children's Hospital of Philadelphia, University of Pennsylvania School of Medicine, Philadelphia, Pennsylvania, United States of America; 6 Division of Allergy, Pulmonary, and Critical Care Medicine, Department of Medicine, Vanderbilt University, Nashville, Tennessee, United States of America; 7 Department of Environmental Health, Harvard School of Public Health and Pulmonary and Critical Care Unit, Department of Medicine, Massachusetts General Hospital, Boston, Massachusetts, United States of America; 8 Cardiovascular Research Institute, Departments of Medicine and Anesthesia, University of California San Francisco, San Francisco, California, United States of America; 9 Department of Surgery, University of California San Francisco, San Francisco, California, United States of America; 10 Department of Genome Sciences, University of Washington, Seattle, Washington, United States of America; 11 Division of Pulmonary, Allergy, and Critical Care Medicine, Johns Hopkins University School of Medicine, Baltimore, Maryland, United States of America; 12 Department of Surgical Sciences, Vanderbilt University, Nashville, Tennessee, United States of America; 13 Division of Oncology, The Children's Hospital of Philadelphia, University of Pennsylvania School of Medicine, Philadelphia, Pennsylvania, United States of America; 14 Division of Pulmonary and Critical Care Medicine, University of Colorado Health Sciences Center, Denver, Colorado, United States of America; Johns Hopkins University, United States of America

## Abstract

Acute Lung Injury (ALI) is a syndrome with high associated mortality characterized by severe hypoxemia and pulmonary infiltrates in patients with critical illness. We conducted the first investigation to use the genome wide association (GWA) approach to identify putative risk variants for ALI. Genome wide genotyping was performed using the Illumina Human Quad 610 BeadChip. We performed a two-stage GWA study followed by a third stage of functional characterization. In the discovery phase (Phase 1), we compared 600 European American trauma-associated ALI cases with 2266 European American population-based controls. We carried forward the top 1% of single nucleotide polymorphisms (SNPs) at p<0.01 to a replication phase (Phase 2) comprised of a nested case-control design sample of 212 trauma-associated ALI cases and 283 at-risk trauma non-ALI controls from ongoing cohort studies. SNPs that replicated at the 0.05 level in Phase 2 were subject to functional validation (Phase 3) using expression quantitative trait loci (eQTL) analyses in stimulated B-lymphoblastoid cell lines (B-LCL) in family trios. 159 SNPs from the discovery phase replicated in Phase 2, including loci with prior evidence for a role in ALI pathogenesis. Functional evaluation of these replicated SNPs revealed rs471931 on 11q13.3 to exert a *cis*-regulatory effect on mRNA expression in the *PPFIA1* gene (p = 0.0021). *PPFIA1* encodes liprin alpha, a protein involved in cell adhesion, integrin expression, and cell-matrix interactions. This study supports the feasibility of future multi-center GWA investigations of ALI risk, and identifies *PPFIA1* as a potential functional candidate ALI risk gene for future research.

## Introduction

Acute lung injury (ALI) is a syndrome characterized by diffuse pulmonary edema and severe hypoxemia in the absence of clinical evidence of left atrial hypertension [1]. ALI affects an estimated 190,000 people annually in the United States and carries a mortality of over 35% [2]. Because only a proportion of patients exposed to predisposing conditions develop ALI (e.g. following sepsis, pneumonia, aspiration, or trauma), it has been hypothesized that individual genetic variation may contribute to the observed variability in ALI susceptibility [3], [4]. Prior studies of genetics of ALI using candidate gene approaches have identified variation in genes controlling inflammation, apoptosis, oxidative stress, or endothelial permeability among others that may confer differential risk of developing ALI [5], [6], [7], [8], [9], [10], [11], [12], [13], [14], [15]. adding to evidence that risk of developing ALI may have a genetic basis.

Genome-wide association studies (GWAS) are powerful, unbiased tools for the identification of common genetic variants, e.g., single nucleotide polymorphisms (SNPs), associated with complex traits [16]. Despite potential limitations [17], GWAS have led to the identification of genetic susceptibility loci that reproducibly confer risk for complex diseases such as Crohn's disease, rheumatoid arthritis, and Type II diabetes [16], [18], [19]. To date, the GWAS approach has not been applied to the study of ALI.

We report the first GWAS of ALI susceptibility, using a three stage approach, including a discovery phase, a replication phase, and a functional evaluation phase using gene expression screening in family trios. We hypothesized that a GWAS approach would identify common genetic variants associated with a reproducible differential risk of ALI.

## Methods

This study was approved by the institutional review boards (IRBs) of the Children's Hospital of Philadelphia (CHOP), the University of Pennsylvania School of Medicine, University of Washington, Harvard School of Public Health, Vanderbilt University, and the University of California at San Francisco.

### Study Populations

We chose an at-risk trauma population to minimize heterogeneity from multiple precipitating factors of ALI and to efficiently allow for comparison with population-based controls, as major trauma is largely a stochastic event across populations. ALI cases and at-risk controls were identified from severe trauma cohort studies performed at 5 U.S. centers: Harvard University/Massachusetts General Hospital, University of Pennsylvania, Vanderbilt University, University of Washington, and University of California at San Francisco. Entry criteria were an Injury Severity Score (ISS) ≥16 and admission through the Emergency department to an intensive care unit. ALI was defined according to American European Consensus Conference (AECC) criteria [20] by trained investigators at each site [13], [14], [21], [22], [23], [24], [25], [26]. Written informed consent was obtained from participants at Harvard University/Massachusetts General Hospital, Vanderbilt University, University of Washington, and University of California at San Francisco. Participants at University of Pennsylvania were collected under a waiver of consent granted by the University of Pennsylvania IRB. Population-based controls were recruited from ongoing cohorts at the Center for Applied Genomics at CHOP [27], [28].

### Study Design

**Figure 1:**
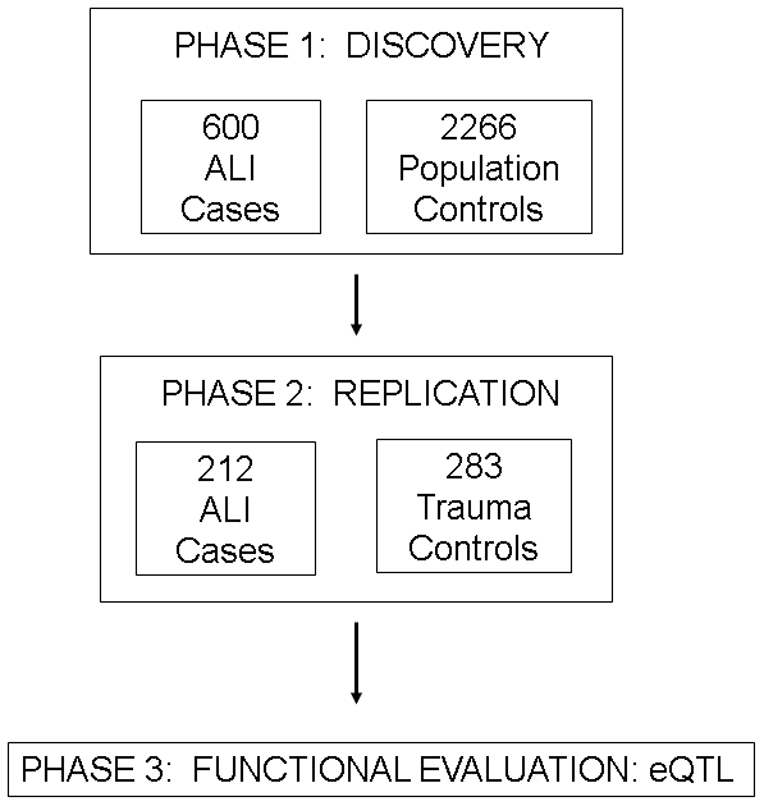
Overview of study design. Abbreviations: ALI, acute lung injury; eQTL, eQTL, expression quantitative trait loci.

We performed a two-stage genotyping strategy [29], with a third stage of functional characterization (Figure 1). In the discovery phase (Phase 1), we compared 600 European American trauma-associated ALI cases with 2266 European American population-based pediatric healthy controls taken from ongoing studies at Children's Hospital of Philadelphia (CHOP). Pediatric population controls [27], [28] were chosen for the discovery phase to maximize efficiency and cost savings [30] for the following reasons: these controls were available in large number and had been genotyped on the same genome-wide platform during a similar time; ALI is not a chronic disease, there is no known effect of cumulative life exposures on ALI risk following trauma; a major environmental insult (trauma) is required to become at risk for ALI; and a recently published study by our group found little difference between these pediatric population controls and other adult controls [31].

We carried forward SNPs with p<0.01 for ALI association in our discovery sample to a replication phase (Phase 2) [29]. The replication population consisted of a nested case-control design of 212 ALI cases and 283 at-risk major trauma controls from ongoing cohort studies at the five participating centers. At-risk controls were defined as severely injured subjects with ISS>16 [32], admitted to the intensive care unit who did not develop ALI during hospital stay, and were primarily drawn from two sites (HSPH and UW). We used logistic regression to adjust for potential confounding clinical factors, including age, ISS, and mechanism of trauma (classified as blunt or penetrating).

SNPs that achieved significance at p<0.05 level in our Phase 2 replication set were subject to functional evaluation using expression quantitative trait loci (eQTL) analyses for *cis*-regulating elements from B-LCL derived from 60 European ancestry and Yoruban HapMap trios (mother, father, offspring) evaluated under two conditions: stimulated with CL097, an innate immune agonist acting through Toll-like receptor 7 (TLR7), or with culture media alone [33]. *Cis*-acting eQTL in the setting of CL097-exposed B-LCL were determined using a linear regression model in unrelated individuals. *Cis*-tests were performed with SNPs (allele frequency>5%) mapping within 1 MB of the array probe start site. *Cis* eQTL were confirmed in the parent-offspring trios using the quantitative transmission-disequilibrium test (QTDT) [34], as implemented in the QTDT software package (http://www.sph.umich.edu/csg/abecasis/QTDT/index.html).

### Genotyping Methods and Quality Control

We used the Illumina HumanQuad610 BeadChip (Illumina, San Diego) to determine genotypes for 620,901 linkage disequilibrium bin-tagging polymorphisms and copy number variation (CNV) markers. Approximately 500 ng of genomic DNA from peripheral blood samples was used for each subject. Each sample was whole-genome amplified, fragmented, precipitated, and resuspended in hybridization buffer. Denatured samples were next hybridized on BeadChips for 16 hours at 48°C, the single base extension reaction performed, and the chip stained and imaged on an Illumina Bead Array Reader. Normalized intensity data for each sample were loaded into Illumina Beadstudio 2.0 and genotypes called using the manufacturer's clustering algorithm. Gender was checked using built-in controls. Clusters were checked for separation, deviation from Hardy-Weinberg, and lack of variation (mono-morphism). Genotypes that did not deviate from Hardy-Weinberg Equilibrium, demonstrated >95% call rate, and a minimum quality score determined by the manufacturer's software, were eligible for statistical association analyses. SNP imputation was also performed on the discovery cohort using the Markov Chain Haplotyping (MACH) software (http://www.sph.umich.edu/csg/abecasis/MaCH/index.html) for genotype imputation on markers that are not present in the genotyping platform used. The software version 1.0.16 was used in the study, and the default two-step procedure was adopted for imputation. The software requires several input files for SNPs and phased haplotypes, so we used the HapMap phased haplotypes (release 22) on HapMap Utah residents with ancestry from northern and western Europe (CEU) subjects, as downloaded from the HapMap database (http://www.hapmap.org). We analyzed the mlinfo file generated from the imputation process and used the recommended R^2^ threshold of 0.3 to flag unreliable markers used in imputation analysis, and removed these markers from association tests. We also analyzed the mlqc file, which provides a per-genotype posterior probability for each imputed call, and we used the 0.9 threshold to flag unreliable calls (by recoding them as NoCall genotype). In total, 2,487,415 imputed autosomal markers (out of 2.54 million markers with phase information in HapMap CEU population) passed quality control for the ALI cohort. We have included all loci with p<0.01 in Phase 1 in Supplemental Table S2.

### Statistical Analysis

Statistical tests for genetic association were conducted using *PLINK* [35]. To reduce the risk of population stratification due to misspecification of self-reported ancestry, we screened all cases and controls at ancestry informative markers (AIMs) using the STRUCTURE software package and removed outliers from principal components analysis. Using the methods of Skol [36] and assuming an additive model, two-stage design, 800 cases, 2500 controls, and carrying forward SNPs with an association p<0.01 in an additive model to stage 2 with a replication alpha of 0.05, we determined that we would have greater than 80% power for both Stage 1 and replication analyses to detect a relative risk of 1.5 or greater for allele frequencies of 0.10 or greater. Power for SNP detection in individual phases was less. Phase 1 with 600 cases and 2200 controls yielded greater than 80% power for a detectable relative risk of 1.5 or greater at an alpha of 0.01 for allele frequencies greater than 0.10 (detectable RR 1.9 for p<5×10^−8^). In Phase 2 we had 80% power to detect relative risks of 1.8 or greater at alpha 0.05 for allele frequencies of greater than 0.10.

## Results

Sample flow of ALI cases is presented in Supplemental Figure S1. Characteristics of the ALI case, population control, and at-risk control populations are contained in Table 1. In Phase 1, the mean age of the ALI cases was 45 years, 70% were male, 92% had blunt trauma and the mean ISS was 27. The mean age of the controls was 9 years and 57% were male. In Phase 2, age, gender, ISS, and mechanism of trauma were similar between cases and controls. Supplemental Table S1 presents subject characteristics by site. Age, gender, and ISS were similar according to individual sites within cases and controls.

**Table 1:** Demographics of Trauma ALI SNP Consortium (TASC) subjects.

	Phase 1 (n = 2866)		Phase 2 (n = 495)	
	Cases (n = 600)	Controls (n = 2266)	Cases (n = 212)	Controls (n = 283)
Gender	n = 596			
Males (%)	419 (70%)	1287 (57%)	158 (75%)	206 (73%)
Female (%)	177 (30%)	979 (43%)	54 (25%)	77 (27%)
Age in years	n = 589	n = 2250	n = 195	n = 270
	44.78 (±20.05)	8.64 (±5.72)	44.32 (±19.66)	38.90 (±20.76)
ISS	n = 542		n = 194	n = 266
	26.97 (±9.98)	NA	27.77 (±10.44)	23.94 (±10.72)
Blunt Injury (%)	n = 528		n = 180	n = 173
	488 (92%)	NA	168 (93%)	149 (86%)

**Abbreviations:** ALI, acute lung injury; SNP, single nucleotide polymorphism; ISS, Injury Severity Score; NA, not applicable.

**Figure 2:**
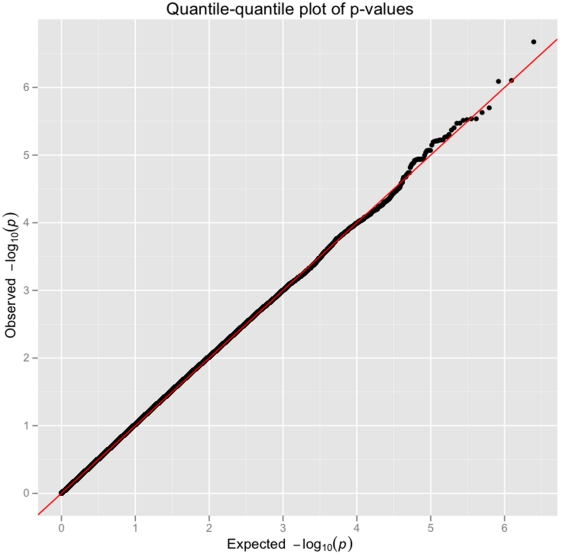
Quantile-Quantile (Q-Q) plot of single SNP association with ALI.

**Figure 3:**
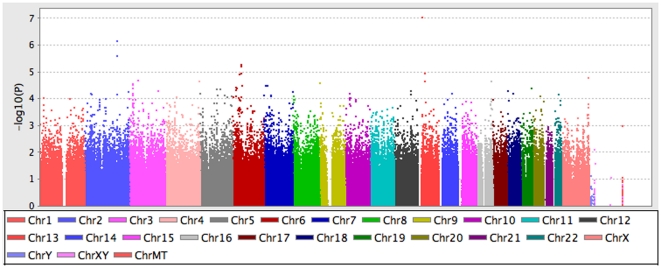
Manhattan plot of −log_10_(p-value) for SNP association with ALI.

Following QC and filtering, the genomic inflation factor for the Phase 1 discovery set was 1.027, indicating minimal differences in underlying population structure between cases and controls (Figure 2). Figure 3 represents a Manhattan plot of −log_10_(p-value) in the Phase 1 discovery cohort. A total of 5815 genotyped SNPs demonstrated association in the Phase 1 discovery cohort at p<0.01; these SNPs were carried forward to the replication phase (Phase 2). Supplemental Table S2 presents results of 28,618 imputed SNPs from Phase 1 that were associated with ALI at p<0.01.

A total of 159 SNPs achieved p<0.05 in the Phase 2 replication set with similar direction of OR when adjusted for age, gender, ISS, and mechanism of trauma (Supplemental Table S3). Of note, several of these SNPS were in genes that could plausibly be associated with mechanisms contributing to ALI (Table 2), including members of the thrombospondin [37], [38], tetraspanin [39], and chitinase families [40], [41]. One replicated variant resulted in an amino acid substitution in the corresponding gene product, a Leu29Ile substitution in CCL24 (or eotaxin-2) [42].

**Table 2:** Replicated SNPS with putative functional roles in ALI pathogenesis.

Chr	SNP	Disc.OR	Disc.p	Riskallele	MAFALI	MAFcontrl	Repl.OR	Repl.p	Symbol	Gene Name	Entrez gene ID
5	rs2398611	0.65	0.0012	C	0.05	0.10	0.47	0.0061	ARHGAP26	Rho GTPase activating protein 26	23092
12	rs1468674	1.25	0.0014	G	0.53	0.46	1.33	0.0386	KLRB1	killer cell lectin-like receptor subfamily B, member 1	3820
5	rs248244	1.60	0.0015	T	0.08	0.05	1.83	0.0347	SQSTM1	sequestosome 1	8878
21	rs2838659	0.78	0.0032	G	0.21	0.25	0.70	0.0330	TSPEAR	thrombospondin-type laminin G domain and EAR repeats	54084
1	rs1321106	1.23	0.0036	C	0.37	0.31	1.41	0.0193	TSPAN2	tetraspanin 2	10100
12	rs2701129	0.69	0.0045	A	0.09	0.13	0.57	0.0179	NR4A1	nuclear receptor subfamily 4, group A, member 1	3164
14	rs847301	0.63	0.0051	T	0.03	0.07	0.47	0.0298	RGS6	regulator of G-protein signaling 6	9628
5	rs4546368	0.82	0.0053	T	0.41	0.47	0.75	0.0371	ABLIM3	actin binding LIM protein family, member 3	22885
7	rs7778918	0.81	0.0055	A	0.30	0.35	0.73	0.0498	THSD7A	thrombospondin, type I, domain containing 7A	221981
10	rs1948837	1.56	0.0061	G	0.06	0.04	1.89	0.0447	PRKG1	protein kinase, cGMP-dependent, type I	5592
8	rs12547884	0.78	0.0068	A	0.18	0.26	0.66	0.0162	MSRA	methionine sulfoxide reductase A	4482
7	rs2302006	0.78	0.0072	A	0.17	0.24	0.70	0.0463	CCL24	chemokine (C-C motif) ligand 24	6369
1	rs3820145	0.78	0.0084	A	0.17	0.23	0.62	0.0104	CHIT1	chitinase 1	1118
10	rs7922288	1.27	0.0086	C	0.20	0.15	1.47	0.0312	PTPRE	protein tyrosine phosphatase, receptor type, E	5791
6	rs237012	1.20	0.0091	A	0.53	0.44	1.35	0.0389	TAB2	TGF-beta activated kinase 1/MAP3K7 binding protein 2	23118
10	rs1317790	1.38	0.0098	T	0.12	0.08	1.61	0.0444	MPP7	membrane protein, palmitoylated 7	143098
3	rs4553956	0.81	0.0101	T	0.22	0.28	0.70	0.0271	TP63	tumor protein p63	8626
8	rs8178179	1.42	0.0107	C	0.08	0.04	2.24	0.0110	PRKDC	protein kinase, DNA-activated, catalytic polypeptide	5591
15	rs10520676	0.80	0.0101	G	0.18	0.24	0.64	0.0109	NTRK3	neurotrophic tyrosine kinase, receptor, type 3	4916

**Abbreviations:** Chr, chromosome; SNP, single nucleotide polymorphism; OR, odds ratio; Disc. or, odds ratio from phase 1 additive trend model; Disc. p, p value from phase 1 additive trend model; maf, minor allele frequency; ali: acute lung injury; repl. or, or from phase 2 additive trend model; repl. p, p value from phase 2 additive trend model.

These 159 SNPs that replicated in Phase 2 were next carried forward to functional evaluation in Phase 3. In eQTL analyses of TLR-7 stimulated HAPMAP B-LCL, there was significant differential expression of PPFIA1 mRNA (p = 0.0021) according to genotype of rs471931, a *cis*-acting SNP on 11q13.3 (Table 3). The *PPFIA1* gene encodes liprin alpha, a protein involved in cell adhesion and cell-matrix interactions [43].

**Table 3:** Replicated SNP that alters expression in stimulated B-lymphoblastic cell lines (B-LCLs) [33].

CHR	SNP	MAFALI/Control	Phase 1 OR	Phase 1 P-value	Phase 2OR	Phase 2P-value	Expressed mRNA	Phase 3 eQTLP-value
11	rs471931	0.50/0.45	1.20	0.0107	1.53	0.0045	PPFIA1	0.0021

**Abbreviations:** SNP, single nucleotide polymorphisms; Chr, chromosome; MAF, minor allele frequency; OR, odds ratio; mRNA, messenger ribonucleic acid; eQTL, expression quantitative trait loci.

We additionally screened our discovery phase to test the association of loci with prior reported association with ALI (Table 4). Notably, the *IL10* SNP (rs1800896 at −1082 in the promoter region) showed a similar effect on ALI risk as in prior reports [12]. However, as our GWA platform was not specifically designed to provide adequate coverage for these loci of interest, many of the previously associated SNPs in other genes were not available. Therefore, we also report results for other SNPs within the same candidate genes in Table 4. Several of these candidate genes contained additional loci associated with ALI risk including *IL10*, *FAS* [44], *MYLK* [45], and *ANGTP2*, although the specific SNPs from prior publications did not replicate in the case of *FAS* and *MYLK*.

**Table 4:** ALI association of previously reported ALI candidate genes at the SNP and gene level in the discovery/Phase I population.

Gene	Chr	SNP	Reference	Candidate SNP	Candidate Gene
F5	1	rs6025 (Arg506Gln)	[63]	N/A	NS
IL10	1	rs1800896 (A/G −1082)	[64]	OR 1.15 p = 0.039	rs1554286
					OR 0.80
					p = 0.00498
IL1RN	2	rs4251961	[65]	OR 1.02 p = 0.80	NS
NFE2L2	2	rs1754059 (C/A −617)	[9]	N/A	NS
SFTPB	2	rs1130866 (T/C +1580)	[66], [67], [68]	OR 1.07 p = 0.54	NS
MYLK	3	rs9840993	[6], [45]	N/A	rs11718105
		rs4678047		OR 0.93 p = 0.33	OR 1.28
		rs28497577		N/A	p = 0.00150
SOD3	4	rs1007991	[7]	N/A	NS
		rs8192291		N/A	
		rs2695232		N/A	
		rs2855262		N/A	
TLR1	4	rs5743551 (A/G −720)	[69]	N/A	NS
TNF	6	rs1800629 (G/A −308)	[70]	OR 0.88 p = 0.48	NS
VEGF	6	rs833061 (C/T −460)	[71], [72]	OR 1.09 p = 0.39	NS
		rs2010963 (C/G +405)		N/A	
		rs3025039 (C/T +936)		N/A	
IL6	7	rs4800795 (G/C −174)	[73], [74], [75], [76]	OR 1.06 p = 0.46	NS
PBEF1	7	rs41496055	[8]	N/A	NS
ANGPT2	8	rs2959811	[77], [78]	OR 0.89 p = 0.10	rs7825407
		rs2515475		OR1.00 p = 0.97	OR 1.27
		rs1868554 – imputed		OR 1.22	P = 0.00189
				p = 0.0083	
FAS	10	rs2147420 - imputed	[44]	OR 0.99 p = 0.86	rs9658691
		rs2234978 - typed		OR .93 p = 0.30	OR 0.68
		rs1051070 - imputed		OR 1.02 p = 0.96	p = 0.00102
MBL2	10	rs1800451	[79], [80], [81]	N/A	NS
PLAU	10	rs1916341	[82]	N/A	NS
		rs2227562		OR 1.02 p = 0.88	
		rs2227564		OR 0.97 p = 0.71	
		rs2227566		N/A	
		rs2227571		N/A	
		rs4065		OR 0.97 p = 0.58	
IRAK3	12	rs10506481 - imputed	[83]	OR 0.96 p = 0.75	NS
NFKBIA	14	rs3138053 (A/G −881)	[84]	OR 0.83 p = 0.073	NS
		rs2233406 (C/T −826)		N/A	
		rs2233409 (C/T −297)		N/A	
HMOX2	16	rs1362626	[85]	OR 0.99 p = 0.87	NS
		rs2404579		N/A	
		rs2270366		OR 1.03 p = 0.70	
		rs1051308		OR 1.03 p = 0.70	
		rs7702		OR 0.97 p = 0.73	
NQO1	16	rs689455	[86]	N/A	NS
FTL	19	rs905238	[85]	OR 1.07 p = 0.31	NS
		rs918546		N/A	
		rs2230267		N/A	
MIF	22	rs2070767	[87]	N/A	NS
		rs755622		N/A	
Structural Variants					
ACE		In/del	[88], [89], [90]	N/A	NS
NFKB1		In/del promoter	[91]	N/A	NS
PAI1		4G/5G	[92]	N/A	NS

SNP-level results are provided if the specific locus previously reported to associate with ALI was either directly genotyped by the Human 660quad platform or was able to be imputed with a posterior probability (r^2^) 0.90. If no imputation was possible due to SNP rarity or lack of linkage disequilibrium with genotyped markers, the result is given as “Not Available” (N/A). At the gene level, the strongest association reported for the gene, as annotated by the NCBI RefSeq position, is reported when associations resulted in a probability *p*≤0.01. If no SNP annotated to the gene was associated with *p*≤0.01, the result is given as “Not Significant” (NS). The results for *ANGPT2* in this population have previously been published [78].

## Discussion

To our knowledge, this is the first genome wide association study to examine the risk of ALI. We demonstrate association at multiple loci in two independent datasets; when coupled to eQTL analysis, the putatively functional results prioritize novel loci for future ALI research. Furthermore, this study provides evidence of feasibility for future GWAS in trauma and other at-risk populations, including those using at-risk controls in the discovery phase.

The putatively functional genetic variant identified in this study suggests a compelling hypothesis to explain the pathogenesis of the observed link with ALI risk. The protein encoded by the *PPFIA1* gene is a member of the liprin (LAR protein-tyrosine phosphatase-interacting protein) family [46]. Liprins have mostly been studied in the nervous system and in mammary gland development [46]; however, liprin alpha binds to the intracellular membrane-distal phosphatase domain of tyrosine phosphatase ‘leukocyte antigen related” (LAR), and may regulate the disassembly of focal cell adhesion, influencing cell-matrix interactions [47]. Liprin alpha has recently been suggested to act by affecting the localization of beta1 integrins [48]. Integrins are involved in the pathogenesis ALI through interactions with the extracellular matrix, altering cell adhesion and lung vascular permeability [49], [50].

The exact function of the genetic regulation of rs471931 on the *PPFIA1* gene is not known. It is approximately 280Kb downstream of *PPFIA1* on 11q13.3 and there are intervening genes, including *SHANK2*. Future studies may focus on fine mapping the region to better understand the role of genetic regulation of liprin alpha expression in ALI risk.

A novel feature of our study is the use of eQTL analyses that employ transformed stimulated lymphocytic cell lines to evaluate functional differences between the genotypes on our list, providing evidence of differential expression according to our ALI-risk genotypes. However, future studies may demonstrate that differential expression exists in cell types that were not included in our approach, such as in pneumocytes or macrophages [18]. Therefore, SNPs not found to have differential expression in our functional phase could potentially have functional consequences in other tissues and be linked to ALI risk. However, as of the time of this publication there were no available datasets with both genome-wide genotyping and genome-wide gene expression in lung specific tissues to our knowledge. As such, we have presented the results of association for the 159 SNPs replicating to nominally significant p-values in Phase 2, as lack of functional correlation in our study does not rule out a role in ALI risk.

Of replicated loci, several have hypothesized putative roles in ALI pathogenesis, as presented in Table 2. In particular, members of the thrombospondin and chitinase families [40], [41] have been implicated in ALI pathogenesis [37], [38]. Tetraspanins have been shown to influence lipopolysaccharide-induced macrophage activation and lung inflammation [39]. CD161 has recently been implicated in regulating the tissue-homing and inflammatory effects of T-cells, including IL-17 producing cells [51], [52], [53]. Methionine sulfoxide reductase A (MSRA) protects against oxidative stress [54], and the *TAB2* gene encodes a protein in the IL-1 signal transduction pathway [55], [56]. *ARGAP26* encodes Rho GTPase activating protein 26; and Rho family GTPases may regulate endothelial barrier integrity through interactions with the actin cytoskeleton [57]. Likewise, the protein product actin-binding LIM 3 (abLIM3) has recently been described to affect barrier integrity in bronchial epithelium through actin binding as a component of the junctional complex [58].

One of our replicated loci resulted in an amino acid change in the protein product CCL24. This encoded protein, also known as eotaxin-2, has been involved in pulmonary allergic response [42], and there is evidence of alteration of levels due to genetic variation in humans [59]. Likewise, the potential functional consequence of the specific IL10 SNP rs1800896 sets this promoter variant as a priority for future studies of ALI. Of note, in the original description by Gong, et al, an interaction was detected with younger age, as is seen in the relatively young age of our trauma study cohort [12]. In addition, although prior published SNPS did not replicate, gene-based replication of prior *FAS*, *ANGTP2*, and *MYLK* associations with ALI prioritize these candidate loci for future study.

The use of population-based controls in the derivation stage allowed for optimization of power while preserving at-risk trauma controls for the replication stage. Although this approach is efficient and economical, there may have been biases introduced because the controls did not suffer the same predisposing risk factor (major trauma), and were drawn from pediatric cohorts. ALI only develops after a severe precipitating insult, and ALI susceptibility genes may be quiescent unless a severe environmental insult occurs (e.g. sepsis, influenza, blood transfusion, or major trauma) [60]. Therefore, it is possible that there are ALI susceptibility genes that were not apparent in our discovery phase (false negative results). Nonetheless, we do not believe our choice of controls in Phase 1 would lead to false positive results for the following reasons: a) none of our candidate genes are plausibly linked with survival to the age of traumatic insult; b) the pediatric population controls have been shown to be similar to adult population controls; and c) and Phase 2 used at-risk nested controls with major trauma that were followed for ALI determination but did not develop the syndrome, thereby minimizing selection bias. However, it is likely that a larger discovery phase using at-risk trauma controls of similar age may uncover additional risk variants. We estimate that 1000 cases and at-risk controls would provide adequate power to detect genotype relative risks greater than 1.6 for common variants, and larger samples would be needed for uncommon variants.

Our study has several additional limitations. First, the discovery sample set is on the lower bounds of power for demonstrating GWAS significance [36], [61]. Although we were powered to detect common SNPs in a two-stage design, our replication sample was modest; we may have missed important (and less common) variants and therefore negative findings should be interpreted with caution [16]. However, our three-stage genotyping approach with functional characterization of the resulting association signals should minimize the chances of our findings being false positive associations. Likewise, for efficiency and economy, our discovery phase used already-genotyped controls from a single site; therefore confounding due to population stratification may have been possible. However, our statistical approach yielded an excellent genomic inflation factor, and our results replicated in subjects taken from several sites in Phase 2, making population admixture unlikely to bias our results. None of our SNPs achieved significance in our derivation set at the genome-wide multiple comparison level (p<5×10^−8^). However, this should not be interpreted as a lack of evidence of a genetic basis for ALI, given the modest sample size, and given the positive results of our three-stage study design [27]. We chose the trauma population to minimize heterogeneity of ALI etiology and efficiently leverage population-based controls in the Phase 1 discovery phase. However, the pathophysiology of ALI following trauma may be different than other at risk ALI populations [62], and therefore the findings may not be generalizable to other causes of ALI. Future studies will need to replicate our findings in other at-risk populations.

As the first GWA in acute lung injury, our study has uncovered an important novel variant regulating *PPFIA1* expression to prioritize for future studies. Furthermore, our study supports the feasibility of using a multiple-staged GWAS approach in future studies of ALI risk. Bench and translational research studies focused on the roles of liprin alpha 1 in ALI pathogenesis seem warranted. In addition, we have replicated a candidate gene with prior ALI association as well as several genes in pathways with evidence of a role in ALI pathogenesis to serve as priority candidates for future study. Like other complex syndromes, additional novel putative risk genes will likely be uncovered with larger discovery sets, as well as GWAS approaches in other at-risk populations, such as sepsis (RC2 HL101779-02).

## Supporting Information

Figure S1 Schematic representation of the quality control method employed. Of the total 1066 cases of ALI submitted for analysis, 217 were excluded from further analyses due to evidence of non-European ancestry. (Abbreviations: ALI, acute lung injury; AIM, ancestry informative markers; PCA, principal components analysis; Pre-QC EA pre-quality control; EA' European-American; GWA, Genome Wide Association).
(TIF)

Table S1 Subjects Characteristics by site. (Abbreviations: ALI, acute lung injury; SNP, single nucleotide polymorphism; ISS, Injury Severity Score; NA, not applicable).
(DOCX)

Table S2 Imputed Phase 1 loci with p<0.01 for ALI association. (Abbreviations: CHR, chromosome; SNP, single nucleotide polymorphism; BP, base pair location; OR, odds ratio; ANNOT, annotated locus).
(XLSX)

Table S3 Phase 2 SNPs with p<0.05 and consistent direction of OR when adjusted for age, gender, injury severity score (ISS), and mechanism of trauma. (Abbreviations: SNP, single nucleotide polymorphism; BP, base pair location OR, odds ratio; Discovery_or_trend, odds ratio from phase 1 additive trend model; Discovery_p_trend, p value from phase 1 additive trend model; maf, minor allele frequency; ali: acute lung injury; replication_or_trend, or from phase 2 additive trend model; replication_p_trend, p value from phase 2 additive trend model; l95_trend, lower bound of the confidence interval for or from phase 2 additive trend model; u95_trend, upper bound of the confidence interval for or from phase 2 additive trend model).
(XLS)
