# Supplementary material for: Genome Wide Association Identifies PPFIA1 as a Candidate Gene for Acute Lung Injury Risk Following Major Trauma
Source: PLoS One. 2012 Jan 25;7(1):e28268. doi: 10.1371/journal.pone.0028268 (PMC3266233; doi:10.1371/journal.pone.0028268)
Supplement: Table S1 — Subjects Characteristics by site. (Abbreviations: ALI, acute lung injury; SNP, single nucleotide polymorphism; ISS, Injury Severity Score; NA, not applicable). (DOCX) [file pone.0028268.s002.docx]

Table 1: Demographics of TASC samples

| **Phase 1 (n=2874)** | | | | | | | |  |
| --- | --- | --- | --- | --- | --- | --- | --- | --- |
|  | Cases (n=600) | | | | | Controls (n=2266) | | |
|  | HPHS (n=26) | UCSF (n=10) | UWash (n=447) | UPenn (n=65) | Vanderbilt (n=52) | |  |  |
| Gender |  |  |  |  |  | |  |  |
| Males (%) | 19 (82.61%) | 6 (60.00%) | 319 (71.85%) | 44 (67.69%) | 25 (52.08%) | | 1287 (56.80%) | |
| Female (%) | 4 (17.39%) | 4 (40.00%) | 125 (28.15%) | 21 (32.31%) | 23 (47.92%) | | 979 (43.20%) | |
| Age in years | 41.39 (±20.93) | 43.1 (±19.23) | 44.48 (±20.17) | 43.04 (±20.55) | 51.79 (±17.11) | | 8.64 (±5.72) | |
| ISS | 28.86 (±10.99) | 30.22 (±9.64) | 26.90 (±10.29) | 26.34 (±7.19) | NA | | NA | |
| Blunt Injury (%) | NA | 9 (90.00%) | 403 (92.43%) | 60 (92.31%) | NA | | NA | |
| **Phase 2 (n=495)** | | | | | | | |  |
|  | Cases (n=212) | | | | | Controls(n=283) | | |
|  | HPHS (n=10) | UCSF (n=4) | UWash (n=163) | UPenn (n=19) | Vanderbilt (n=16) | | HPHS (n=110) | UWash (n=173) |
| Gender |  |  |  |  |  | |  |  |
| Males (%) | 5 (55.56%) | 3 (75.00%) | 126 (77.78%) | 14 (73.68%) | 7 (52.85%) | | 66 (67.35%) | 132 (76.74%) |
| Female (%) | 4 (44.44%) | 1 (25.00%) | 36 (22.22%) | 5 (26.32%) | 6 (47.15%) | | 32 (32.65%) | 40 (23.26%) |
| Age in years | 45.00 (±22.49) | 45.75 (±20.17) | 44.86 (±19.72) | 38.21 (±18.42) | 54.92 (±17.66) | | 26.32 (±12.17) | 33.84 (±18.96) |
| ISS | 34.11 (±10.67) | 41.5 (±12.87) | 19.72 (±10.29) | 28.79 (±8.53) | 29.13 (13.79) | | NA | 22.65 (±9.66) |
| Blunt Injury (%) | NA | 3 (75.00%) | 147 (93.63%) | 18 (94.74%) | NA | | NA | 149 (86.13%) |
